# External validation of the 4C mortality score among COVID-19 patients admitted to hospital in Ontario, Canada: a retrospective study

**DOI:** 10.1038/s41598-021-97332-1

**Published:** 2021-09-20

**Authors:** Aaron Jones, Tyler Pitre, Mats Junek, Jessica Kapralik, Rina Patel, Edward Feng, Laura Dawson, Jennifer L. Y. Tsang, MyLinh Duong, Terence Ho, Marla K. Beauchamp, Andrew P. Costa, Rebecca Kruisselbrink, the COREG Investigators, William Ciccotelli, Sophie Corriveau, George Farjou, Stephen Giilck, Carla Girolametto, Lauren Griffith, Brent Guy, Shariq Haider, Rajendar Hanmiah, Paul Hosek, Cindy Cin Yee Law, Theresa T. Liu, Maura Marcucci, Leslie Martin, John Neary, Ameen Patel, Natya Raghavan, Parminder Raina, Samir Raza, Connie Schumacher, Catherine Tong, Joshua Wald

**Affiliations:** 1 Department of Health Research Methods, Evidence, and Impact, McMaster University, 1280 Main St W, Hamilton, ON L8S 4L8 Canada; 2 Waterloo Regional Campus, Michael G. DeGroote School of Medicine, McMaster University, Hamilton, Canada; 3 Department of Medicine, McMaster University, Hamilton, Canada; 4 Niagara Health, St. Catharines, Canada; 5 School of Rehabilitation Science, McMaster University, Hamilton, Canada; 6 Centre for Integrated Care, St. Joseph’s Health System, Hamilton, Canada

**Keywords:** Prognosis, Infectious diseases

## Abstract

Risk prediction scores are important tools to support clinical decision-making for patients with coronavirus disease (COVID-19). The objective of this paper was to validate the 4C mortality score, originally developed in the United Kingdom, for a Canadian population, and to examine its performance over time. We conducted an external validation study within a registry of COVID-19 positive hospital admissions in the Kitchener-Waterloo and Hamilton regions of southern Ontario between March 4, 2020 and June 13, 2021. We examined the validity of the 4C score to prognosticate in-hospital mortality using the area under the receiver operating characteristic curve (AUC) with 95% confidence intervals calculated via bootstrapping. The study included 959 individuals, of whom 224 (23.4%) died in-hospital. Median age was 72 years and 524 individuals (55%) were male. The AUC of the 4C score was 0.77, 95% confidence interval 0.79–0.87. Overall mortality rates across the pre-defined risk groups were 0% (Low), 8.0% (Intermediate), 27.2% (High), and 54.2% (Very High). Wave 1, 2 and 3 values of the AUC were 0.81 (0.76, 0.86), 0.74 (0.69, 0.80), and 0.76 (0.69, 0.83) respectively. The 4C score is a valid tool to prognosticate mortality from COVID-19 in Canadian hospitals and can be used to prioritize care and resources for patients at greatest risk of death.

## Introduction

Coronavirus disease (COVID-19) caused by the Severe Acute Respiratory Syndrome Virus 2 (SARS-CoV-2) can progress to acute respiratory distress syndrome, multiorgan failure, and death in some individuals^1^. The COVID-19 infection-fatality risk varies widely with age, with Canadian estimates ranging from < 0.1% for children and younger adults to 25.7% for adults 80 and older^2^. The clinical presentation and progression of COVID-19 in patients is highly variable^3^, which makes it difficult for clinicians to triage patients and determine their risk of poor outcomes. While some patients may clearly present with severe disease, even patients presenting with mild symptoms may experience rapid decompensation^4^. A simple, validated prognostic tool utilizing data that is available at presentation can help clinicians better prognosticate and make clinical decisions. Numerous tools to predict mortality in COVID-19 patients have been developed, but many are limited due to small derivation cohort sizes and/or inadequate validation^5^.

The 4C mortality score is an accessible risk stratification score developed by the International Severe Acute Respiratory and Emerging Infections Consortium (ISARIC)^6^. It was derived and internally validated on a large, diverse cohort within the United Kingdom but requires external validity to confirm its generalizability. The objective of this study was to validate the ability of the 4C score to prognosticate mortality in COVID-19 patients admitted to hospital in Ontario, Canada. We will also examine the performance of the score over time as there has been significant variation in in available treatments, vaccine uptake, and dominant viral lineages over the course of the pandemic.

## Methods

### Study design and setting

We conducted a retrospective validation study using records from the McMaster Multi-Regional Hospital Coronavirus Registry (COREG). COREG is a multi-center data registry collecting information on positive COVID-19 cases in the Kitchener-Waterloo and Hamilton regions of southern Ontario, Canada. The registry includes COVID-19-related emergency department (ED) visits and hospital admissions from six tertiary hospitals across the regions, including three academic and three community centres. The total number of inpatient beds across all sites is approximately 2400.

### Participants

We selected all patients admitted to one of the participating hospitals with a positive SARS-CoV-2 PCR nasal swab test between March 4, 2020 and June 13, 2021. We set the end date on the inclusion window to four weeks before date of final data extraction to allow for at least a 28-day length of stay for all participants. All inpatients were tested for COVID-19 therefore asymptomatic patients admitted for other reasons were included. Elective procedures however required a negative COVID test and therefore were not included. If a patient was transferred between study sites or had two separate admissions only the latter admission was retained. For temporal analysis, patients were separated into waves based on their admission date. Wave 1 spanned March 4, 2020 to August 31, 2020, Wave 2 included dates from September 1, 2020 to February 28, 2021 and Wave 3 stretched from March 1, 2021 to June 13, 2021.

### Measures

Data in COREG were manually abstracted from electronic medical records by trained abstractors using a modified case report form published by ISARIC and the World Health Organization. Data was regularly audited to ensure accuracy. We utilized demographic and clinical data at presentation, typically from the emergency department. For patients who were directly admitted, we utilized the first day of inpatient records. Supplementary Table S1 includes additional information on data collection procedures.

### 4C mortality score

The 4C mortality score was derived and validated within the ISARIC World Health Organization Clinical Characterisation Protocol UK study^7^. The score was derived from a population of over 35,000 hospital inpatients validation on over 22,000 inpatient records indicated good discriminability (area under the receiver operating characteristic curve (AUC) = 0.77)^6^.

The 4C score incorporates age, sex, comorbidities, respiratory rate, peripheral oxygen saturation, Glasgow Coma Scale, blood urea nitrogen, and C-reactive protein. We adapted the score to match our available data as the Glasgow Coma Scale was not collected at presentation. We replaced this risk factor with the documented presence of altered consciousness or confusion. The 4C score ranges from 0 to 21 with risk groups defined as Low (0–3), Intermediate (4–8), High (9–14), and Very high (≥ 15).

### Outcome

The primary outcome was all-cause in-hospital mortality. Patients who received a palliative discharge were included. As was done in the initial derivation paper, we did not place a limit on time until death.

### Statistical analysis

We presented a demographic and clinical profile of study participants, stratified by outcome, and reported the prevalence and missingness of each of element of the 4C score. Missing data was treated by multiple imputation with chained questions with predictive mean matching, using 20 imputations and 50 iterations per imputation. The imputation model included all variables in the 4C score, site id, and outcome. We plotted the proportion of patients who died in hospital by 4C score and risk group and compared them to the initial derivation work in the UK.

We validated the 4C score using the AUC, with 95% confidence intervals calculated via bootstrapping with 2000 resamples. For comparative purposes, we also calculated the AUC using only the age components of the 4C score as age has consistently been shown to be among the strongest predictors of COVID-19 related mortality^8^ and can be easily collected and used to triage patients without electronic aid. We calculated all AUCs overall and by wave. Additionally, we calculated diagnostic accuracy measures for cut-offs at scores of 3, 8, 12, and 14. These cut-offs align with the predefined risk groups with an additional group within the “High” category as half of our study participants were classified within this group. Finally, we constructed a calibration plot using bootstrapped predicted probabilities. All analysis was done in R 4.0.3^9^.

### Ethics approval

Our study received ethics approval from the Tri-hospital Research Ethics Board and the Hamilton Integrated Research Ethics Board, who waived the requirement for informed consent as the data for this study was retrospectively collected from hospital medical records. All methods were performed in accordance with relevant guidelines and regulations.

## Results

Our study included 959 patients of which 224 (23%) died (Table 1, Supplementary Table S2). Median age was 72 with slightly more males (55%) than females. Wave 2 was the largest wave with 46% of patients, followed by wave 1 at 32% and wave 3 at 22%. The missingness and post-imputation prevalence of each element of the 4C score can be found in Table 2. Missingness ranged from 0% for age to 57% for c-reactive protein.

**Table 1:** Characteristics of COVID-positive hospital inpatients and emergency department patients in the Kitchener-Waterloo region of Canada, March 2020 – January 2021.

Characteristic^a^	Overall	Died	Survived
	n = 959	n = 224	n = 735
Age, years	72 (59, 83)	82 (74, 88)	69 (56, 80)
Sex, males	524 (55)	124 (55)	400 (54)
**Region**			
Kitchener-Waterloo	354 (37)	86 (38)	268 (36)
Hamilton	605 (63)	138 (62)	467 (64)
**Wave**			
1. March–August 2020	307 (32)	75 (33)	232 (32)
2. September 2021–February 2021	444 (46)	111 (50)	333 (45)
3. March 2021–June 2021	208 (22)	38 (17)	170 (23)
**Presenting symptoms**			
Temperature (C)	37.0 (36.5,37.9)	37.0 (36.5, 37.8)	37.0 (36.5,38.0)
Cough	551 (57)	102 (46)	449 (61)
Shortness of breath	551 (57)	111 (50)	440 (60)
Fatigue	431 (45)	84 (38)	347 (47)
Altered consciousness/Confusion	218 (23)	89 (40)	129 (18)
Heart rate, per minute	91 (78, 106)	89 (74, 106)	92 (79, 106)
Respiratory rate, per minute	20 (18, 24)	20 (18, 24)	20 (18, 24)
Oxygen saturation, room air	95 (92, 97)	94 (92, 96)	95 (93, 97)
**Comorbidities**			
Chronic lung disease^b^	184 (18)	63 (28)	161 (22)
Chronic cardiac disease	171 (18)	62 (28)	109 (15)
Diabetes	343 (36)	84 (38)	259 (35)
Chronic liver disease	31 (3)	9 (4)	22 (3)
Chronic kidney disease	132 (14)	49 (22)	83 (11)
Chronic neurological disease	118 (12)	29 (13)	89 (12)
Cancer	143 (15)	53 (24)	90 (12)
Obesity^c^	106 (11)	15 (7)	91 (12)
Rheumatologic disease	90 (9)	24 (11)	66 (9)
Dementia	132 (14)	50 (22)	82 (11)
HIV/AIDs	< 6	< 6	< 6
Number of comorbidities	1 (0, 2)	2 (1, 3)	1 (0, 2)
**Biomarkers**			
Blood urea nitrogen, mmol/L	7.5 (5.0, 12.0)	11.0 (7.1, 14.9)	6.7 (4.6, 10.1)
C-reactive protein, mg/L	72 (32, 151)	105 (51, 159)	66 (26, 141)
White blood cells, × 10^9^/L	6.9 (5.0, 9.5)	7.5 (5.6, 11.2)	6.6 (4.9, 9.0)
Platelets, × 10^9^/L,	205 (162, 269)	208 (149, 262)	203 (164, 271)
Neutrophils, × 10^9^/L	5.0 (3.4, 7.3)	5.6 (3.8, 8.6)	4.8 (3.3, 7.0)
Lymphocytes, × 10^9^/L	0.90 (0.60, 1.30)	0.80 (0.6, 1.3)	0.90 (0.60, 1.30)
**4C risk groups**			
Low (0–3)	75 (8)	0 (0)	75 (10)
Intermediate (4–8)	251 (26)	20 (9)	231 (31)
High (9–14)	516 (54)	141 (63)	375 (51)
Very high (15–21)	117 (12)	63 (28)	54 (7)
**Route of admission**			
Emergency department	896 (93)	212 (95)	684 (93)
Transfer/direct	63 (7)	12 (5)	51 (7)

^a^Categorical variables presented as n, (%). Continuous variables presented as median (q1, q3).

^b^Does not include asthma.

^c^As defined by staff.

**Table 2:** Components of the 4C mortality score, missingness, and post-imputation prevalence.

Variable		4C mortality score points	%Missing	Post-imputation prevalence (%)
**Age (years)**			0.0	
< 50		–		12.7
50–59		2		12.4
60–69		4		18.6
70–79		6		23.1
≥ 80		7		33.2
**Sex**			0.1	
Female		–		45.3
Male		1		54.7
**Number of comorbidities**^**a**^			0.4	
0		–		25.7
1		1		30.2
≥ 2		2		44.1
**Respiratory rate (per minute)**			13.5	
< 20		–		39.5
20–29		1		48.6
≥ 30		2		11.9
**Peripheral oxygen saturation (%)**			6.9	
≥ 92		–		82.0
< 92		2		18.0
**Altered consciousness/confusion**			2.2	
No		–		
Yes		2		23.2
**Blood urea nitrogen (mmol/L)**			11.6	
< 7		–		46.5
7–14		1		35.9
≥ 14		3		17.5
**C–reactive protein (mg/L)**			57.0	
< 50		–		38.3
50–99		1		22.0
≥ 100		2		39.7

Adapted from https://www.bmj.com/content/370/bmj.m3339.

^a^Includes chronic lung disease (excluding asthma), chronic cardiac disease, diabetes, chronic liver disease, chronic kidney disease, chronic neurological disease, cancer, obesity (as defined by staff), rheumatological disease, dementia, and HIV/AIDS.

### Validation

**Figure 1:**
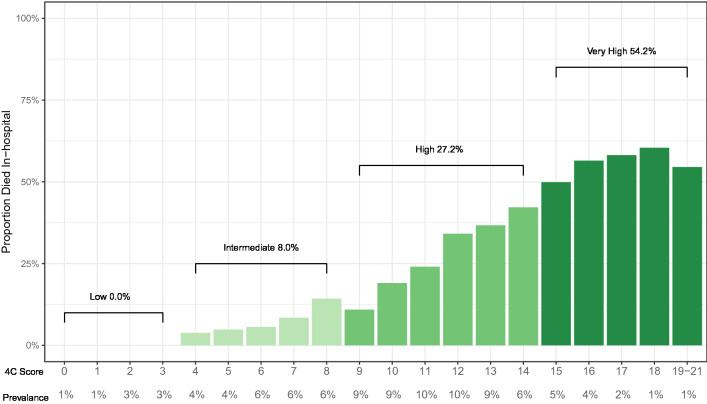
Proportion of COVID-19 inpatients who died by 4C mortality score and risk group.

The plot of the proportion of patients who died in each risk score demonstrated a clear upwards trend (Fig. 1). Mortality rates within the risk groups were 0% (Low), 8.0% (Intermediate), 27.2% (High), and 54.2% (Very High). These mortality rates are similar to the rates reported in the original research (1.2%, 9.9%, 31.4%, and 61.5%)^6^.

The AUC of the 4C score across all patients was 0.77, 95% CI (0.74, 0.80) (Table 3). The performance of the model was higher in wave 1 (0.81 (0.76, 0.86) than wave 2 (0.74 (0.69, 0.80)) or wave 3 (0.76 (0.69, 0.83), although confidence intervals overlapped. The age-only model was somewhat less discriminative than the full model in each wave: wave 1 (0.78 (0.72, 0.83)), wave 2 (0.68 (0.63, 0.73)), wave 3 (0.74 (0.67, 0.80)).

**Table 3:** Area under the receiver operating characteristic curve (AUC) for 4C score and age-only model overall and by wave.

AUC	Full 4C score	Age-only model
Overall	0.77 (0.74, 0.80)	0.73 (0.70, 0.76)
Wave 1	0.81 (0.76, 0.86)	0.78 (0.72, 0.83)
Wave 2	0.74 (0.69, 0.80)	0.68 (0.63, 0.73)
Wave 3	0.76 (0.69, 0.83)	0.74 (0.67, 0.80)

Diagnostic accuracy measures for cut-offs at 3, 8, 11, and 14 can be found in Table 4. The sensitivity of each cut-off ranged from 100% for > 3 to 28.3% for > 14 while the specificities ranged from 10.2% for > 3 to 92.7% for > 14. The calibration plot (Supplementary Figure S3) indicated good calibration with significant deviations only occurring at very high predicted probabilities where data was scarce.

**Table 4:** Diagnostic accuracy measures for mortality prediction at several cut-offs of the 4C score.

4C cut-off	Prevalence (%)	Sensitivity (%)	Specificity (%)	Positive predictive value (%)	Negative predictive value (%)	Positive likelihood ratio	Negative likelihood ratio
> 3	92.2	100.0	10.2	25.3	100.0	1.11	0.00
> 8	66.0	91.0	41.6	32.2	93.8	1.56	0.22
> 11	37.7	68.9	71.8	42.7	88.3	2.44	0.43
> 14	12.2	28.3	92.7	54.2	80.9	3.89	0.77

## Interpretation

We found that the 4C mortality score is a valid tool to prognosticate mortality among COVID-19 patients admission to hospitals in a Canadian population. We observed an overall AUC of 0.77, which is identical to the initial derivation research^6^. The AUC values across waves 1, 2, and 3 were 0.81, 0.74 and 0.76, respectively. The AUC of the 4C score was higher than an age-only model across each of the three waves.

The 4C score has been validated in jurisdictions outside of the United Kingdom, including Canada^10,11^. Our study is confirmatory of these findings and additionally contributes to the literature by conducting an analysis by wave to investigate changes in the performance of the score over time. Changes over time in treatment practices (e.g. use of steroids), vaccine distribution, and the dominant strain of SARS-CoV-2 could all potentially impact the predictive ability of a mortality risk score. The 4C score was derived during the initial phases of the pandemic, before variants of concern emerged, before steroids were demonstrated effective against severe disease^12^, and while vaccines were still in early trials. While these conditions are similar to wave 1 in Ontario, by the peak of wave 2 vaccine distribution was underway to individuals most at risk^13^ and the alpha variant (B.1.1.7) was spreading^14^.

Overall, the predictive ability of the 4C was maintained across the three waves, which is a promising indication given that COVID variants are likely to continue to emerge^15^. We observed a drop in the point estimate of the discriminative ability of 4C score in the later waves, although the differences were not statistically significant. Both the 4C score and age-only model had higher AUC values for wave 3 than wave 2, which may be due to the targeting of early vaccine distribution to older individuals, which would have muted the effect of age on mortality. Future research should examine if the score continues to be predictive as vaccine uptake reaches herd immunity levels.

The proliferation of COVID-19 risk models is evidence of the demand for an accurate, accessible, and generalizable tool^16^, and our research adds to the body of evidence supporting use of the 4C score. Our examination of cut-offs within the 4C score suggests that in practice the score may ultimately be most useful in identifying individuals at particularly low risk of death. The lower two cut-offs (3 and 8) demonstrated negative likelihood ratios of 0 and 0.2, while positive likelihood ratios for any cut-off never exceeded 4. Automated calculation of the 4C score in electronic medical records could be used guide resource management and support clinical decision making such as treatment initiation and admission to ICU.

### Limitations

A key limitation of our study is that we were only able to include data from two regions within southern Ontario. While similarities between health systems across Canada suggest our findings will have excellent generalizability to other Canadian provinces and territories, our results may not generalize to geographically remote settings or to jurisdictions with substantially different health systems. Also, certain variables had high levels of missingness. Although we used multiple imputation per best practice^17^, model performance may have been nevertheless adversely affected by data missing not at random. Additionally, we were not able to collect and report data on race and ethnicity. Finally, the scores were calculated retrospectively and not in real-time.

## Conclusion

The 4C mortality score is an valid prognostic tool for use in Canadian hospitals. It can be used to identify and prioritize care for COVID-19 patients at high risk of death.

## Supplementary Information

Supplementary Information.

## Data Availability

The data used in this study can be accessed for research purposes by submitting a request through the COREG data access portal (https://www.coregontario.ca/info-data-access).

## Code availability

Code is available upon request.
