# Supplementary material for: External validation of the 4C mortality score among COVID-19 patients admitted to hospital in Ontario, Canada: a retrospective study
Source: Sci Rep. 2021 Sep 20;11:18638. doi: 10.1038/s41598-021-97332-1 (PMC8452633; doi:10.1038/s41598-021-97332-1)

Supplementary Information for:

External validation of the 4C mortality score among COVID-19 patients admitted to hospital in Ontario, Canada: a retrospective study

Aaron Jones[1,2], PhD*
Tyler Pitre[3], MD
Mats Junek[3], MD
Jessica Kapralik[3], MD
Rina Patel[2], BSc
Edward Feng[2], BSc
Laura Dawson[2], BSc
Jennifer LY Tsang[3,4], MD, PhD
MyLinh Duong[3], MD,
Terence Ho[3], MD,
Marla K. Beauchamp[5,3], PhD,
Andrew P. Costa[1,3], PhD,
Rebecca Kruisselbrink[3,2]MD,
on behalf of the COREG Investigators[#]

[#]William Ciccotelli, Sophie Corriveau, George Farjou, Stephen Giilck, Carla Girolametto, Lauren Griffith, Brent Guy, Shariq Haider, Rajendar Hanmiah, Paul Hosek, Cindy Cin Yee Law, Theresa T. Liu, Maura Marcucci, Leslie Martin, John Neary, Ameen Patel, Natya Raghavan, Parminder Raina, Samir Raza, Connie Schumacher, Catherine Tong, and Joshua Wald

Includes:

1. Supplementary Table S1– Data collection procedures
2. Supplementary Table S2 - Characteristics of COVID-positive hospital inpatients and emergency department patients in the Kitchener-Waterloo region of Canada, March 2020 – January 2021 (Row percentages for categorical variables)
3. Supplementary Figure S3 – 4C calibration plot using bootstrapped predicted probabilities

Supplementary Table S1 – Data collection procedures

RedCAP: All data for the COREG study was collected on a secure REDCAP system, that was based on the ISARIC-SPRINT SARI REDCAP form. It was modified to fit the structural requirements of data collection locally and to answer pre-determined scientific questions. Please refer to the data library for full details of the REDCAP form. While small differences existed between collection forms used at each institution reflecting additional research priorities, all institutions' data could still be easily merged. The REDCAP form has robust data quality checks that were utilized to flag abnormal entries and to audit missing data.

Data collection manual and practices The data collection activities for each site were coordinated by a local operations lead. The leads liaised with site electronic medical record (EMR) experts to develop a data collection manual that standardized an approach to data collection, and constructed a data dictionary such that there was a consensus method for collecting each data point. Regular operations lead meetings were held to adjudicate disputes in data collection practices, agree on ambiguous terms, and standardize data points.

Data collectors and training: Data collectors included residents and medical students from McMaster University. At the Kitchener-Waterloo site, 14 medical students participated. In Hamilton, there were two medical students and two residents for Hamilton Health Sciences and two residents and 1 medical student for St Joseph's Healthcare. Each of these students underwent training sessions for data collection, which addressed use of the EMR, the contents of the data collection manual, and best practices. Individual practice cases were then completed to ensure consistency of data collection. The same practice case was assigned to all new collectors and thereafter were checked by the operations leads for consistency. This was done twice in Kitchener-Waterloo and with three cases in Hamilton and Niagara. Operations leads then reviewed the data collection and addressed common issues in large group sessions and also focused on specific collectors who needed additional support on an individual basis.

Data Collection: Data was collected remotely. Each data collector was only able to access cases assigned to them by their local operations lead, and only able to see a given patient's institutional identification number to minimize threats to privacy. Collectors were instructed to flag any items they were unsure of and wanted operation lead review. Operations leads made regular audits of the data by validating entered data against patient records as well as auditing summary results to minimize inconsistencies.

Patient selection: Each institution had a separate method of determining COVID-19 patients at their respective institutions. In the Kitchener-Waterloo region, the operations lead received large deposits of COVID-19 positive patients through the EMR's flagging system. This list was divided into three categories of COVID-19 patients: screening only, emergency department visit with discharge home and admission to hospital. Screening only patients refer to patients who were tested at the Grand River Hospital or Saint Mary's General Hospital outpatient screening programs. This list of patients only included pre-existing information on the EMR about past medical history and lab work. Emergency room visit MRNs included patients who went to the emergency room for symptoms and were found to be COVID-19 positive. They differ from screening only in that they were admitted to the emergency room, there is a formal physician examination, assessment and lab and radiological assessment. Inpatients are either patients admitted to the emergency room and therefer admitted to hospital for COVID-19 or inpatients admitted for another reason who then developed nosocomial COVID-19.

In Hamilton Health Sciences, only inpatient data was collected. COVID-19 patients were cared for by a separate team where daily census lists were available. These lists were checked regularly and their patients added to the registry; a separate validation was performed by comparing all patients to a list of all individuals with positive COVID-19 tests at Hamilton Health Sciences as recorded by the Infection Prevention and Control team. Patients who were admitted to the Juravinski Hospital were transferred to the Hamilton General Hospital as soon as their diagnosis was confirmed; transfers were confirmed and duplicates removed by comparing patient census lists.

In St. Joseph's Healthcare, the emergency room and inpatient data were collected. COVID-19 patients were identified by the Infection Prevention and Control (IPAC) service and maintained on a list within the EMR to facilitate care and monitoring. Through collaboration with IPAC access to this list was obtained. This list was reviewed twice weekly for identification of new COVID-19 positive patients for inclusion.

Units: Data collected in the REDCAP form did not include units, only integer values for blood work was included. Instead, each case is pinned to an institution with a list of the blood work units associated with it. This system avoids data collectors erroneously recording a false unit to a specific lab value, decreases the number of data values collected and facilitates interinstitutional comparisons

Supplementary Table S2: Characteristics of COVID-positive hospital inpatients and emergency department patients in the Kitchener-Waterloo region of Canada, March 2020 – January 2021 (Row percentages for categorical variables)

Characteristic[1]	Died n=224	Survived n=735
**Region**		
Kitchener-Waterloo	86 (24)	268 (76)
Hamilton	138 (23)	467 (77)
**Wave**		
1 - March - August 2020	75 (24)	232 (76)
2 -September 2021 - February 2021	111 (25)	333 (75)
3 - March 2021 - June 2021	38 (18)	170 (82)
**Comorbidities**		
Chronic lung disease[2]	63 (28)	161 (72)
Chronic cardiac disease	62 (36)	109 (64)
Diabetes	84 (24)	259 (76)
Chronic liver disease	9 (29)	22 (71)
Chronic kidney disease	49 (37)	83 (63)
Chronic neurological disease	29 (25)	89 (75)
Cancer	53 (37)	90 (63)
Obesity[3]	15 (14)	91 (86)
Rheumatologic disease	24 (27)	66 (73)
Dementia	50 (38)	82 (62)
HIV /AIDs	<6	<6
**4C Risk Groups**		
Low (0-3)	0 (0)	75 (100)
Intermediate (4-8)	20 (8)	231 (92)
High (9-14)	141 (27)	375 (73)
Very High (15-21)	63 (54)	54 (46)
**Route of admission**		
Emergency Department	212 (24)	684 (76)
Transfer/Direct	12 (19)	51 (81)

1. Categorical variables presented by n (%)
2. Does not include asthma
3. As defined by staff

Supplementary Figure S3 – 4C calibration plot using bootstrapped predicted probabilities

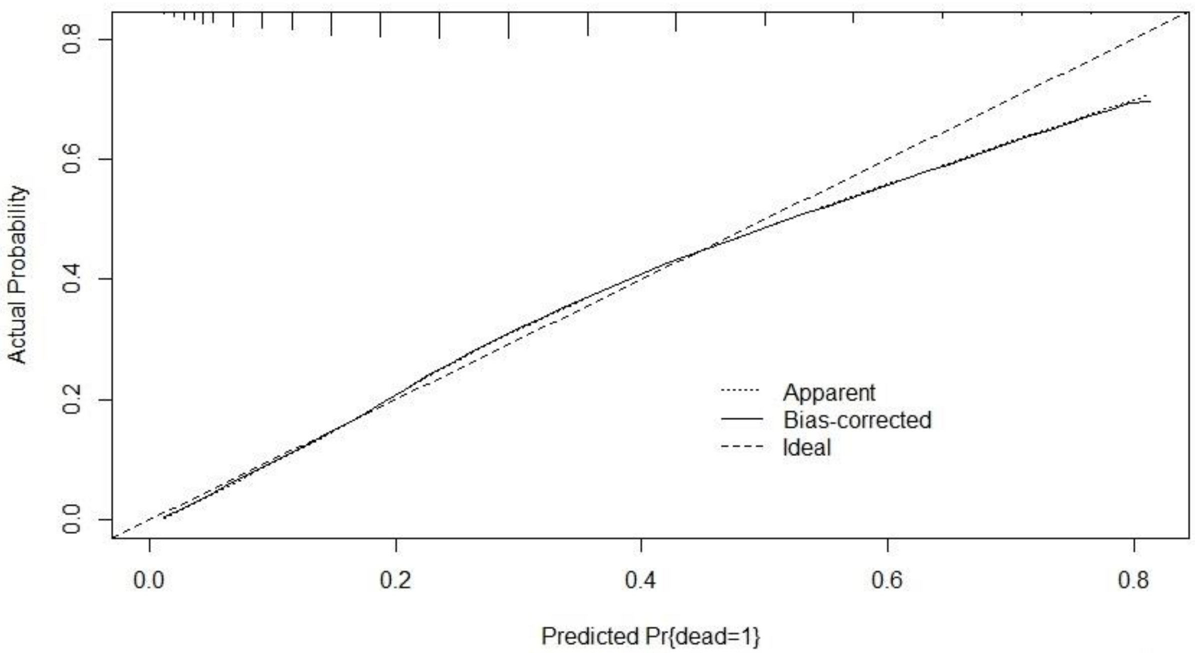

Supplement: Supplementary file 1 — Supplementary Information. [file 41598_2021_97332_MOESM1_ESM.pdf]
